# Natural logarithm particle swarm optimization for loss reduction in an island power system

**DOI:** 10.1016/j.mex.2024.102924

**Published:** 2024-08-20

**Authors:** Alessandra F. Picanço, Antônio C. Zambroni de Souza, Andressa Pereira Oliveira

**Affiliations:** a Department of Automation and System, Federal Institute of Bahia (IFBA), Salvador, Bahia, Brazil; b Federal University of Itajubá (UNIFEI), Inst. of Electrical System and Energy, Itajubá, Minas Gerais, Brazil; c Collegiate of Electrical Engineering, Federal University of Western Bahia (UFOB), Bom Jesus da Lapa, Bahia, Brazil

**Keywords:** Contingency, Distributed generation, Distribution power system, Optimal allocation, Radial power system, Natural Logarithm PSO (NLogPSO)

## Abstract

In an island power system, optimizing energy management is fundamental since there are renewable sources with their limitations. This management includes the allocation and capacity of energy sources to supply the loads. In this context, optimizing losses in the system contributes to improve the efficiency of this management. This paper proposes the losses optimization and energy management in the island power system. The authors propose the Natural Logarithm Particle Swarm Optimization to solve the problem and compare it with the Attractor Point Algorithm and Evolutionary Particle Swarm Optimization. And with that, we also propose a particle initialization for the studied particle-based algorithms to guarantee convergence in radial power systems. This is because the system configuration influences the response of the algorithm convergence. These techniques were applied to the IEEE-34 unbalanced radial island system.

• Natural Logarithm Particle Swarm Optimization differs from classical PSO in that it does not calculate the velocity of the particles. Therefore, the method considers a cloud of particles with a natural logarithmic trajectory to solve the reduction of losses in a power system with a radial topology.
• Natural Logarithmic Particle Swarm Optimization uses an initialization equation to minimize the initial estimation process, which is relevant to the convergence process.

## Graphical abstract

**Figure:**
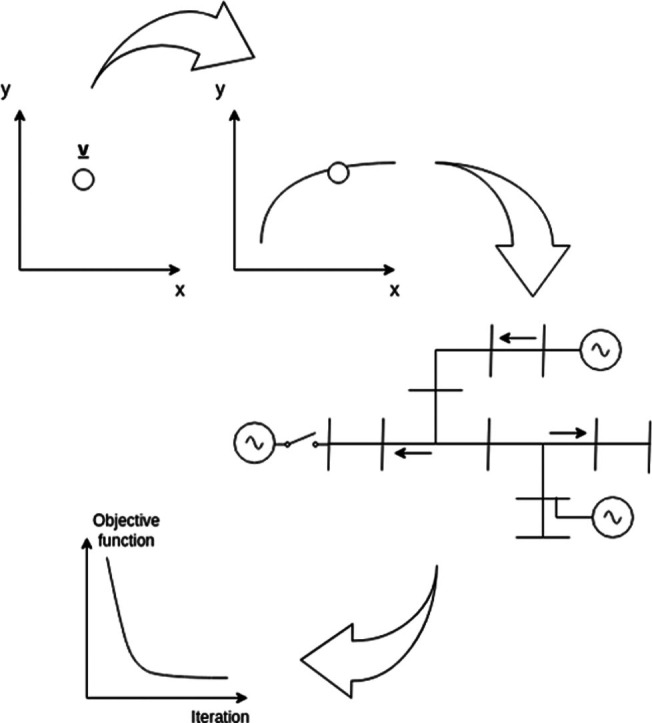

Specifications table

**Table:** 

Subject area:	Engineering
More specific subject area:	*Electrical and Energy*
Name of your method:	*Natural Logarithm PSO (NLogPSO)*
Name and reference of original method:	*N.A.*
Resource availability:	IEEE-34 Node Test Feeder (https://site.ieee.org/pes-testfeeders/resources/)

## Background

Renewable Energy Sources (RES), such as solar, wind, and other micro-sources, can be sustainable, flexible and accessible. However, the growing application of distributed generations and electric vehicles, which characterizes the Distributed Energy Resource (DER), requires a more efficient operation of the elements in the system. Therefore, the microgrid (MG) controls DERs and loads, maximizing efficiency between generation and demand, whether connected to the grid or island mode [1]. Isolated microgrids have renewable energy generators, distributed generation (DG), battery energy storage systems (BESS) and loads. Due to the uncertainty scenarios of MG agents, there is an optimization of the energy management strategy (EMS) [[2], [3], [4]].

Energy storage devices have been widely used in loss reduction, expansion deferral, island operation, and voltage control applications. Mobile generators and MESS (Mobile Energy Storage Systems) can be used in an isolated mode to maintain and restore the distribution system, such as the reducing operating costs in a 33-bus distribution system and voltage regulation through reactive power support [5,6]. Restoration services with mobile resources can occur due to the mobile emergency generators' predicted positioning or real-time allocation. The Renewable Mobile Power Station (RMPS) associated with MG can guarantee energy supply in case of a disconnection [7] and with the PSO (particle swarm optimization) method can be used for its optimal allocation [8]. Probabilistic methodology was used to optimize the location and capacity of wind and solar generation in island mode for 33-bus radial system [9]. And in the context of loss reduction, a heuristic distributed algorithm was developed to calculate the radial power flow of an island operation and optimize losses in the 9-bus and 25-bus system [10]. As shown in Table 1, the problem solved involves optimizing the operational cost, energy management, allocation and optimal generation capacity, or power flow with the study of the harmonic content generated by the DGs.

**Table 1:** Points covered in the cited references.

References	Optimization of EMS	Optimal allocation/capacity of mobile generators in the island system	Minimization of losses in the island system	Harmonic power flow in the island system/with DG
[[1], [2], [3], [4],^11^,^12^]	Yes	No	No	No
[[5], [6], [7], [8], [9],^13^]	Yes	Yes	No	No
[10]	No	No	Yes	No
[[14], [15], [16]]	No	No	No	Yes

PSO-based algorithms have been successfully applied to power system optimization. An example of application in radial power systems is the analysis of voltage profile and stability index. For the case of multiple DGs, enhanced PSO and Ant-Lion optimization were applied to a multi-objective function, and the results between the algorithms were similar [17]. The application of PSO was compared with Grey Wolf Optimization Algorithm and Whale Optimization Algorithm for loss reduction and optimal DG allocation in an IEEE-33 bus radial system with similar results [18]. Novel PSO (NPSO) was proposed to reduce losses and optimise voltage deviation in an IEEE-33 bus system. The proposed method created a new solution search space using the cosine function to calculate the inertia weight to disperse the particles [19]. Another possible modification to the standard PSO was to add a new part to the particle velocity equation to reduce losses in IEEE-9 and IEEE-14 bus systems [20]. Hybrid algorithms based on PSO applied to electrical systems have been proposed by some authors [[21], [22], [23], [24]].

RMPS can work with DG to supply power in an island system due to a contingency. In this case, the objective is to optimize the RMPS location to attend priority loads, which the EMS will define. This paper aims to improve the efficiency of an island system with a harmonic source through a combined optimization of the RMPS capacity and loss reduction. Therefore, we propose the Natural Logarithm PSO algorithm (NLogPSO) to solve this problem. The difference is that NLogPSO uses the particle position in the trajectory based on the natural logarithm to explore the search space of solutions, so it is not necessary to calculate the particle velocity. For example, during the NLogPSO tests on the benchmark functions, the *gbest* of 4.36 × 10^–5^ was obtained for $F_{1}\left(x\right)=\sum_{i=1}^{n}x_{i}^{2}$ with 100 particles in the interval [−100,100], a function similar to the calculation of losses in the radial power system. For comparison purposes, this study employs the attractor point algorithm (APA) that works in two layers: the main layer minimizes losses, and the second determines the optimal capacity of the RMPS. In this context, the two-layer APA algorithm is compared with the two-layer Evolutionary Self-Adaptative Particle Swarm Optimization (EPSO). The APA and EPSO algorithms are combined to form two hybrid algorithms: APA-EPSO and EPSO-APA to evaluate their performance. As the initial particles influence the convergence, we also propose an equation for initializing the particles in optimizing the radial power flow. Loss reduction is obtained by considering the temperature of the conductors and harmonic sources in the Backward-Forward Sweep (BFS) power flow to provide more variables and thus increase numerical complexity. These results, therefore, change the autonomy capacity of the microgrid. It is worth noting that temperature-dependent power flow for unbalanced radial systems [25] considers the Carson model [26], the BFS algorithm, and the application of temperature-dependent resistances of conductors in the power flow, in which it is regarded the heat transfer processes in the conductors from the current and environmental conditions, such as wind speed, wind angle, solar radiation, and temperature [Anon., 27]. In this case, the state variables are the voltage magnitude, voltage angle, and temperature of the conductors. The main contribution is the NLogPSO algorithm, which allows one to reach the following goals, described as:

• *Losses minimization in an island system:* the presence of harmonic sources can increase losses in the system. Considering the calculation of the temperature of the conductors contributes to improving the accuracy of the losses. Therefore, the addition of these factors as variables makes it difficult for the algorithms to converge and one of the objectives of this paper is to consider these variables in the optimization process.
• *Optimal allocation of RMPS:* to solve the problem of a programmed disconnection in a system, this paper applied the LSF method [20], so that the allocation of RMPS contributes to loss minimization. For this, two conditions are proposed for the application of this method.
• *Particle-based optimization:* for the optimization of losses in the islanded system for a determined number of hours and optimal energy management provided by the RMPS.
• *PSO enhancement*: the proposed methodology used APA, EPSO and NLogPSO in two layers. This paper also proposes the particle initialization equation to minimize system losses and optimize the energy supplied by the RMPS.

Then, NLogPSO, APA, EPSO, APA-EPSO, and EPSO-APA are applied in three cases using the unbalanced 34-bus radial system.

## Method details

### Optimal allocation of RMPS

In this paper, the buses were selected for DG allocation in an island system based on the temperature-dependent power flow [25], while considering the impact on the survival time of the island system. Optimal RMPS allocation was determined by the active loss sensitivity factor (LSF) method according to (1) [28]. The proposed application consists of allocating the largest capacity generation in the first bus listed by the method. The location of the other generations, if their capabilities are less than the first, must follow the priority listed by the LSF method regardless of their capability. MGs are considered PQ models in power flow. In summary, this paper proposes the LSF method with the following statements:

• *Statement 1: Place the highest capacity RMPS or DG on the first bus indicated by the LSF method.*
• *Statement 2: RMPS that are not in statement 1 can be allocated to any bus indicated by the LSF method regardless of its capacity.*
  (1) $$LSF=\frac{\partial P_{L}}{\partial P_{s}}=2\sum_{j=1}^{N}\left(\alpha_{st}P_{t}-\beta_{st}Q_{t}\right)$$

Where,

(2) $$P_{L}=\sum_{s=1}^{N}\sum_{t=1}^{N}\left[\alpha_{st}\left(P_{s}P_{t}+Q_{s}Q_{t}\right)+\beta_{st}\left(Q_{s}P_{t}-P_{s}Q_{t}\right)\right]$$

(3) $$\alpha_{st}=\frac{r_{st}\left(T\right)}{V_{s}V_{t}}cos\left(\delta_{s}-\delta_{t}\right)$$

(4) $$\beta_{st}=\frac{r_{st}\left(T\right)}{V_{s}V_{t}}sin\left(\delta_{s}-\delta_{t}\right)$$

*P_s_, P_t_* are the active powers in bus *s* and *t; Q_s_, Q_t_* are the reactive powers in bus *s* and *t; V_s_, V_t_* are the voltage magnitudes in bus *s* and *t;δ_s_, δ_t_* are the voltage angles in bus *s* and *t*. Finally, *N* is the bus number in the system. LSF values are calculated for all buses and arranged in descending order, thus establishing a list of candidate buses for RMPS allocation.

### Island system optimization

Optimizing the island system with RMPS is carried out in two stages: loss minimization (first layer) and adequacy of RMPS capacity (second layer). The power flow optimization problem consists of a non-linear objective function with non-linear constraints. The objective function is given by (5).

(5) $$f_{PF}\left(x\right)=min\left(f_{loss}+f_{v}+f_{P}+f_{Q}\right)$$

Where,

(6) $$f_{loss}=\sum_{s=1}^{N}\sum_{t=1}^{N-1}\left[r_{st}\left(T\right)\right]xI_{st}^{2}$$

(7) $$f_{v}=c_{\rho }\sum_{s=1}^{N}{\left[max\left(V_{s}-V_{max}\right)\right]}^{2}+c_{\rho }\sum_{s=1}^{N}{\left[max\left(V_{min}-V_{s}\right)\right]}^{2}$$

(8) $$f_{P}=c_{\rho }\sum_{s=1}^{N}{\left[P_{G,s}-P_{d,s}-\sum_{t=1}^{N-1}\left[r_{st}\left(T\right)\times I_{st}^{2}\right]\right]}^{2}$$

(9) $$f_{Q}=c_{\rho }\sum_{s=1}^{N}{\left[Q_{G,s}-Q_{d,s}-\sum_{t=1}^{N-1}\left[x_{st}\times I_{st}^{2}\right]\right]}^{2}$$

*r_st_(T)* and *x_st_* are the *st* branch temperature-resistance and reactance, *V_min_* and *V_max_* are the minimum and maximum voltage equal to 0.9 and 1.1 pu, respectively, *P_G,s_* is the active power generation in the bus *s, Q_G,s_* is the reactive power generation in the bus *s, P_d,s_* is the active power demand in the bus *s* and *Q_d,s_* is the reactive power demand in the bus *s* and *c_ρ_* is the penalty coefficient.

The algorithm applied in this paper works in two layers. The first layer considers branch currents as particles in PSO-based optimization algorithms, and the secondary layer is the optimization of the RMPS generation capacity to serve the island system.

### Evolutionary PSO optimization

Evolutionary Self-Adaptative Particle Swarm Optimization (EPSO) [29] considers the learning parameter *τ* in the weights applied to the particle velocity and the best global particle. Therefore, the particle position and velocity are calculated according to (10) and (11), respectively. The best global particle *gbest’* is updated in (12), where *rand* is the random number with Gaussian distribution within the range [0,1]. In this paper, w_i0_=rand and w_i1_=w_i2_=2*,* where considered.

(10) $$x_{i}^{\left(k+1\right)}=x_{i}^{\left(k\right)}+v_{i}^{\left(k+1\right)}$$

(11) $$v_{i}^{\left(k+1\right)}=w_{i0}.v_{i}^{\left(k\right)}+w_{i1}.\left(pbest_{i}-x_{i}^{\left(k\right)}\right)+w_{i2}.\left(gbest^{\prime }-x_{i}^{\left(k\right)}\right)$$

(12) $$gbest^{\prime }=gbest+\tau .rand$$

### Attractor point algorithm

The particles converge to their attractor point (13) with coordinates in (14) in quantum-behaved particle swarm optimization (QPSO) [30,31].

(13) $$p_{i}^{\left(k\right)}=\left[p_{i,1}^{\left(k\right)},p_{i,2}^{\left(k\right)},...\ldots ,p_{i,n}^{\left(k\right)}\right]$$

(14) $$p_{i,j}^{\left(k\right)}=\frac{c_{1}.u_{i,j}^{\left(k\right)}.pbest_{ij}^{\left(k\right)}+c_{2}.U_{i,j}^{\left(k\right)}.gbest_{i}^{\left(k\right)}}{c_{1}.u_{i,j}^{\left(k\right)}+c_{2}.U_{i,j}^{\left(k\right)}}$$

where *U_i,j_* and *u_i,j_* are random numbers with Gaussian distribution within the range [0,1]. *c_1_* and *c_2_* are the acceleration factors equal to 2, *pbest* is the best position of particle and *gbest* is the best global position, *k* is the iteration and *n* is the total number of particles.

The particle's position in the QPSO is updated according to (15).

(15) $$x_{i,j}^{\left(k\right)}=p_{i,j}^{\left(k\right)}\pm \kappa .\left|C_{i}^{\left(k\right)}-x_{i,j}^{\left(k-1\right)}\right|.ln\left(1/rand\right)$$

Where *C* is known as the *mbest* position, it is the average of the *pbest* positions of all particles and *κ* is the contraction-expansion coefficient [30,31].

This paper proposes calculating the particle's position only with the attractor point of the radial system (16). Therefore, it is not necessary to use Eq. (15) for updating the position of the QPSO particle. The second part of (15) does not influence the result, regardless of the value applied. Thus, we will use the name attractor point algorithm (APA) as a special case of QPSO.

(16) $$x_{i,j}^{\left(k\right)}=p_{i,j}^{\left(k\right)}$$

In radial power flow, the particles are the branch currents that must be checked within the *I_max_* and *I_min_* constraints produced by the temperature-BFS algorithm according to (17). Equality constraints are used when phases are not used in the single-phase branch as in (18).

(17) $$I_{min}\leq x_{i,j}^{\left(k\right)}\leq I_{max}$$

(18) $$x_{i,j}^{\left(k\right)}\left(phase\right)=0$$

### Natural logarithm PSO

This paper proposes the Natural Logarithm PSO (NLogPSO) for optimization of radial power systems, where the particles are based on the natural logarithm and updated according to (19). The Eq. (19) yields a set of logarithmic trajectories as a search space for the solution. The natural logarithm of *rand* was used, since the number *e* is present in many natural processes. The *rand* is a random number between 0 and 1 based on the Gaussian distribution.

(19) $$x_{i,j}^{\left(k+1\right)}=ln\left(rand\right).\left[x_{i,j}^{\left(k\right)}-\left(gbest_{i}-pbest_{i,j}\right)\right]$$

In the EPSO particle velocity equation, three weights have random numbers; in the APA equation, there are two weights and two acceleration factors. NLogPSO's proposal is to apply only one weight to its equation, which gives the particle natural behavior through ln*(rand)*.

From a framework, EPSO calculates the difference between the *pbest* and the most recent particle, which is added in the particle update. This also happens with *gbest* and the weights are multiplied by these differences. In the case of APA, the new particles are calculated with the values of *pbest* and *gbest* at each iteration. And the particle update in NLogPSO is performed by subtracting the most recent particle and the difference between the *gbest* and *pbest* of the set, and the result is inserted in a natural logarithm behavior.

### Secondary layer: DG/RMPS capacity optimization

The DG/RMPS capacity optimization problem is a non-linear objective function with non-linear constraints. The objective function is given in (20).

(20) $$f\left(x,z,t_{m}\right)=min\left(\sum_{i=1}^{N_{G}}Pg_{i}-f_{loss}-P_{L}^{\left(l\right)}\left(t\right)\right)$$

Where *x* is the system vector state variables, *z* is the DGs vector and *t_m_* is the system time in island mode, *Pg* is the power supplied, *N_G_* is the number of generations, *f_loss_* are the system losses and *P_L_* is the load power.

In case the DG/RMPS capacity is not enough to supply the load, the consumers must be classified according to their load priority. The highest priority load is indicated by 1 and the lowest priority by 3 [7]. Medical assistance, telecommunications and public lighting are high priority consumers (*l* = 1). Industrial and commercial are *l* = 2 and, residential and rural, *l* = 3. In this case, the load power is calculated as in (21).

(21) $$P_{L}^{\left(l\right)}\left(t\right)=\sum_{i=1}^{N}\sum_{t_{m}=t_{isl}}^{t_{prog}}\varphi_{i}^{\left(l\right)}\left(i,t_{m}\right)P_{L,i}^{\left(l\right)}\left(t_{m}\right)$$

The operational status matrix $\varphi_{i}^{\langle l\rangle }\left(i,t_{m}\right)$ is related to load priority according to bus and time. The operational status assumes the value 0 for loads off and 1, for loads on. *N* is the bus, *t_isl_* is the initial system time and *t_prog_* is the time programmed in island mode.

Particle restrictions are checked within each energy source's minimum and maximum power limits (22). The installed generation can be wind, solar, or battery.

(22) $$P_{min}\leq y_{i,j}^{\left(k\right)}\leq P_{max}$$

The second layer optimization can be performed by the EPSO or APA algorithms. For APA, the particles *y_i,j_* converge to the attractor point in the same way as in (13) and (14), resulting in (23). Therefore, the attractor point algorithm was used to optimize the DG/RMPS capacity.

(23) $$y_{i,j}^{\left(k\right)}=Pg_{i,j}^{\left(k\right)}$$

### Initial particles

Particle-based optimization algorithms demand a collection of initial particles, as with EPSO and APA. Therefore, the determination of initial particles plays a key role in convergence. In the case of loss reduction optimization, these particles use the branch current of the BFS-temperature power flow. For the radial topology of the system, this paper proposed the calculation of the initial particles according to (24) and used in the EPSO, APA and NLogPSO algorithms.

(24) $$x_{i,j}^{\left(0\right)}=I_{st}-\left(I_{max}-I_{min}\right)\times \left[\frac{-ln\left(rand\right)}{\lambda }\right]$$

Where *i* represents the variable position, *j* is the indication of the particle, *I_st_* is the current in the branch in cartesian coordinates, *rand* is the random number in the interval [0,1] with Gaussian distribution, *I_max_* is the maximum current and *I_min_* is the minimum current calculated by the temperature-BFS radial power flow for the island system, λ∈R.

The use of λ in the initial particles is to help the convergence of the algorithm through its adjustment. This is because in some situations, increasing the number of particles does not produce good results and the algorithm can stall around an undesired solution.

The proposed initial particles are calculated according to (25) for the secondary layer.

(25) $$y_{i,j}^{\left(0\right)}=Pg_{i,j}-rand\times \left(P_{max,i}-P_{min,i}\right)\times \left[\frac{-ln\left(rand\right)}{\lambda }\right]$$

Where *y_i,j_^(0)^* is the initial particle, *Pg_i_* is the nominal power generation *i, P_max,i_* is the maximum power generation *i, P_min,i_* is the minimum power generation *i, rand* is the random number in the range [0,1] with Gaussian distribution. *Δt* is the time interval in hours and $\lambda_{\Delta t}>0$.

### Algorithms

**Fig. 1:**
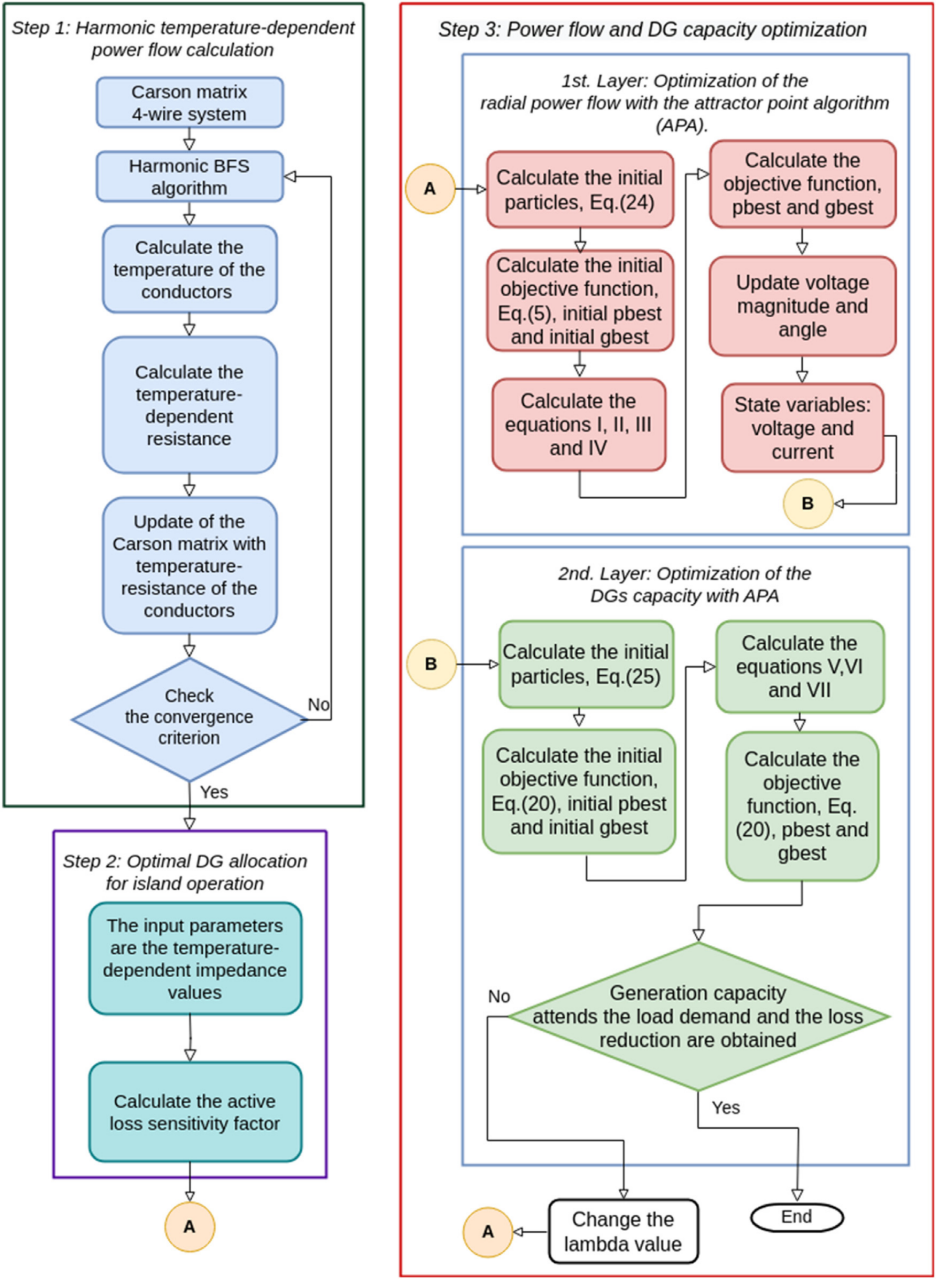
Algorithm flowchart with harmonic temperature-dependent power flow calculation, optimal DG allocation for island operation and power flow and DG capacity optimization.

Fig. 1 presents the algorithm flowchart in its three main steps. In step 1, the system's temperature-dependent power flow with harmonics is calculated. The power flow data calculates the optimal allocation points of the DGs/RMPS in step 2. And in step 3, the power flow is calculated for the island system with the DGs/RMPS, which consists of optimizing losses and DGs/RMPS capacity depending on the algorithm used: APA, EPSO or NLogPSO. The flowchart in Fig. 1 shows equations I – VII, which are different in the EPSO, APA and NLogPSO algorithms. Thus, Table 2 presents each algorithm's equations used in the flowchart.

**Table 2:** Equations I-VII of the flowchart in Fig. 1.

Algorithms	Equations						
	I	II	III	IV	V	VI	VII
APA-APA	(14)	(16)	(17)	(18)	(23)		(22)
APA-EPSO					(10)	(11)	
EPSO-EPSO	(10)	(11)			(10)	(11)	
EPSO-APA					(23)		
NLogPSO—NLogPSO	(19)				(19)		

## Method validation

**Fig. 2:**
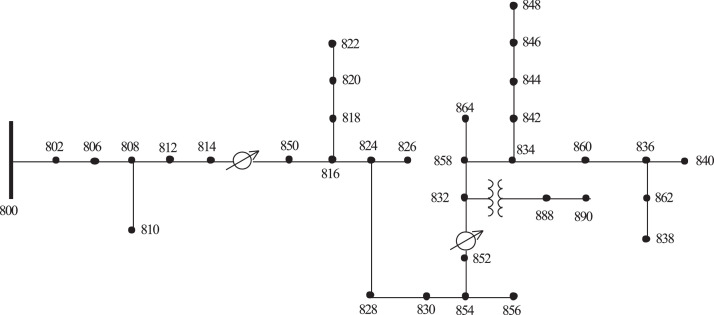
IEEE-34 unbalanced radial bus system [29].

The algorithm was developed in MATLAB© computing environment using Intel® Core™ i5–8265 U CPU @ 1.60 GHz and 8 GB of RAM. It was applied to the IEEE-34 unbalanced radial bus system depicted in Fig. 2 [Anon., 32]. The network configuration data, three-phase and single-phase, and the spacing and height of the cables necessary to calculate the Carson model, were collected in reference [Anon., 32]. The method used to calculate the power flow was Backward-Forward Sweep [26] considering the temperature of the conductors and the harmonic content (Harmonic Temperature-BFS method). Wind speeds of 0.2 and 2.0 m s^-1^ were considered with an inclination of 45° and conductor type 428-A1/S1A-54/7‘ZEBRA’ [Anon., 27].

The IEEE-34 system was employed to simulate three load demands and was isolated from the main grid for 4 h. For the island system of cases 1 and 2, the optimal location of the energy sources using LSF method was *N_RPMS_* = {890,830,844}. For case 3, the optimal location of the energy sources was *N_RPMS_* = {890,830,844,860}. The LSF method indicates the list of buses for optimal RMPS allocation. The first bus on this list must be allocated to the RMPS with the largest capacity, as proposed in statement 1. RMPS that do not follow statement 1 may be allocated to any bus indicated in the LSF list. The installed power in case 1 is 1.45 MW, in case 2 is 1.59 MW, and in case 3 is 2.50 MW.

Two harmonic sources were randomly inserted: an induction motor at bus 828 and a VFD (6-pulse variable frequency drive) at bus 816. The power flow analysis considered the influence of this harmonic content on the branches adjacent to the buses of these loads. Thus, the feeders influenced by the harmonic current were (824, 828), (828, 830), (816, 824), (850, 816) and (816, 818). In these branches, the skin effect due to the harmonic content was considered. The VFD has a active power of 120 kW and a reactive power of 80 kVAr. The 6-pole induction motor has a shaft power of 229.5 kW, a power factor of 0.853 and an efficiency of 97.5 %. Table 3 presents the current spectra of VFD [16] and induction motor [33,34].

**Table 3:** Current spectra of VFD and induction motor.

Current spectra of VFD [14].			Current spectra of induction motor [Anon., 32].	
Harmonic order	Amplitude (%)	Phase (°)	Harmonic Order	Amplitude (%)
1	100	0	1	100
5	18.24	−55.68	5	3.0
7	11.9	−84.11	7	2.5
11	5.73	−143.56	11	1.5
13	4.01	−175.58	13	1.0
17	1.93	111.39	17	0.2
19	1.39	68.30		

The method validation is divided into two parts. The first part shows the results of the proposed algorithms optimizations for harmonic temperature-BFS. The second one deals with considerations about priority loads.

### Method validation I: algorithms optimization

The initial particles are calculated according to (24) and are essential for the convergence process. For that reason, it was necessary to establish a numerical region where λvalues would be decisive. It was observed that to obtain loss reduction, the absolute value of $x_{i,j}^{\left(0\right)}$ must be <0.5. Thus, λ should be chosen to produce $x_{i,j}^{\left(0\right)}\leq 0.5$.However, the optimal loss reduction value is obtained with $0.4\leq x_{i,j}^{\left(0\right)}\leq 0.5$. To minimize losses in the objective function (5) a penalty coefficient of 10^–3^ was used and tolerance in the algorithms was 10^–3^.

**Fig. 3:**
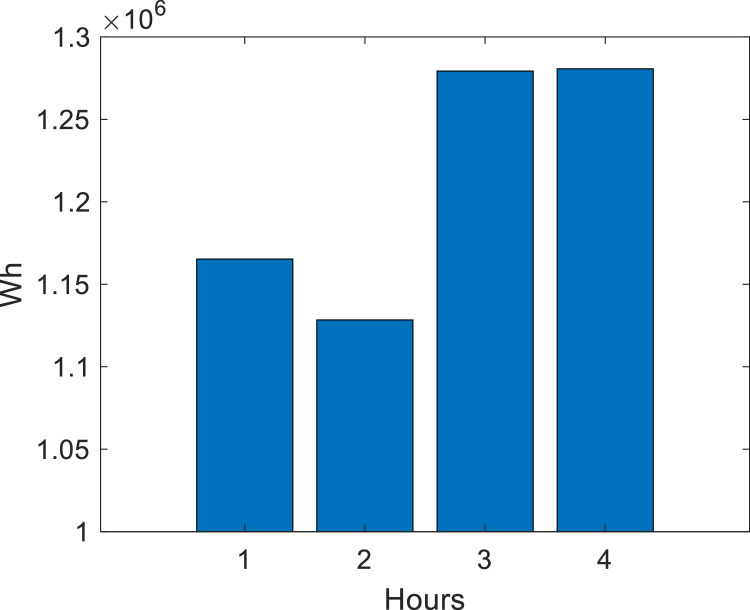
Load power demand for case 1.

For **case 1**, three RMPS are considered, where the maximum generation power at bus 890 is 1300 kW per phase, 60 kW per phase at bus 830 and 90 kW per phase at bus 844, *P_RMPS_* = {1300,60,90} kW in the buses *N_RPMS_* = {890,830,844}. In addition, the induction motor at bus 828 influences the branch (824,828) due to the skin effect. Fig. 3 shows the load power demand during island operation for case 1.

**Fig. 4:**
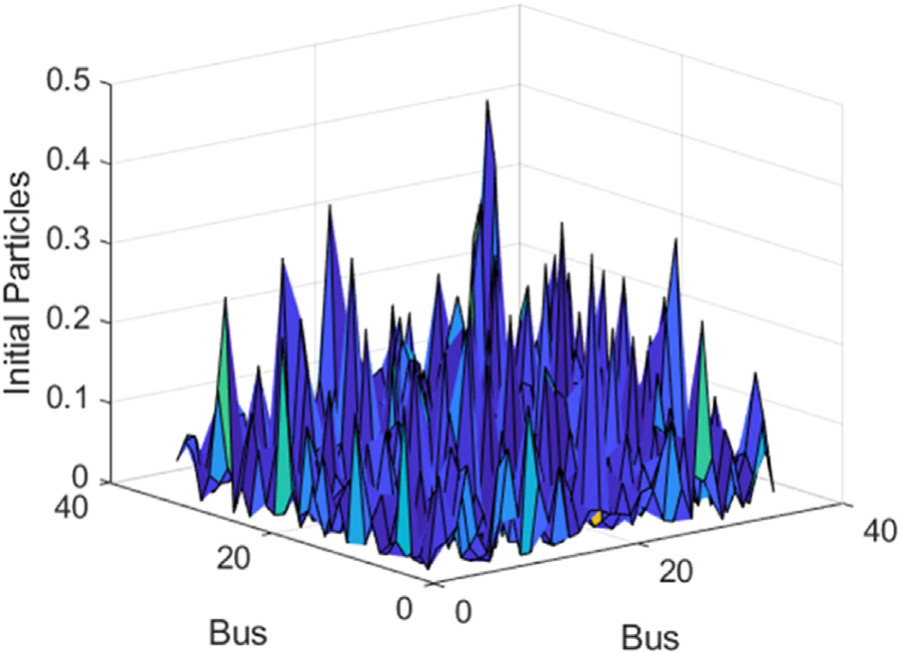
Initial values surface for λ=20 and resulted of APA-APA algorithm.

For APA-APA, EPSO-EPSO and NLogPSO—NLogPSO algorithms, the optimal values were obtained with 1000 particles for loss minimization in the harmonic temperature power flow. The optimal λ for loss reduction was 20 and for optimal capacity of DG was 2. Fig. 4 presents a surface of initial values for λ=20.

Table 4, Table 5 present the results of active and reactive power considering a wind speed of 0.2 m s^-1^ and 2.0 m s^-1^, respectively, in the harmonic temperature-BFS algorithm without optimization and with EPSO-EPSO, APA-APA and NLogPSO—NLogPSO algorithms. The proposed optimization algorithms reduce losses at the same levels for both wind speeds.

**Table 4:** Results of active and reactive power with harmonic temperature-BFS, EPSO-EPSO, APA-APA and NLogPSO—NLogPSO algorithms for the wind speed of 0.2 m s^-1^.

Hour	kW	Harmonic Temperature-BFS			EPSO-EPSO			APA-APA			NLogPSO—NLogPSO		
	kVAr	A	B	C	A	B	C	A	B	C	A	B	C
1	P	52.0	11.1	23.5	48.2	4.7	9.4	48.4	8.9	15.5	48.4	4.2	4.9
	Q	31.8	6.5	16.3	27.7	2.6	4.6	27.8	5.3	9.5	27.8	2.6	3.2
2	P	52.0	11.1	23.5	49.0	4.8	9.4	48.5	8.9	15.5	48.5	4.3	4.9
	Q	31.8	6.6	16.3	28.3	2.6	4.7	27.8	5.3	9.6	27.8	2.6	3.2
3	P	52.1	11.3	23.6	49.1	4.9	9.6	48.5	9.1	15.6	48.5	4.3	5.0
	Q	31.9	6.6	16.4	28.4	2.7	4.7	27.9	5.4	9.6	27.9	2.6	3.2
4	P	52.2	11.3	23.7	49.1	5.0	9.6	48.6	9.1	15.7	48.6	4.3	5.0
	Q	31.9	6.7	16.4	28.4	2.7	4.8	27.9	5.4	9.7	27.9	2.6	3.2
Mean active power reduction (%)					6.19	56.70	59.70	6.86	19.64	33.93	6.86	61.60	79.00
Mean reactive power reduction (%)					11.46	59.84	71.25	12.55	18.93	41.13	12.56	60.60	80.42

**Table 5:** Results of active and reactive power with harmonic temperature-BFS, EPSO-EPSO, APA-APA and NLogPSO—NLogPSO algorithms for the wind speed of 2.0 m s^-1^.

Hour	kW	Harmonic Temperature-BFS			EPSO-EPSO			APA-APA			NLogPSO- NLogPSO		
	kVAr	A	B	C	A	B	C	A	B	C	A	B	C
1	P	50.5	10.8	22.8	46.2	4.5	8.9	45.8	8.4	14.6	45.7	4.1	4.7
	Q	31.8	6.5	16.3	28.3	2.6	4.6	27.8	5.3	9.5	27.8	2.6	3.2
2	P	50.5	10.8	22.8	46.3	4.5	8.9	45.8	8.4	14.6	45.8	4.1	4.7
	Q	31.8	6.6	16.3	28.3	2.6	4.7	27.8	5.3	9.6	27.8	2.6	3.2
3	P	50.6	10.9	22.9	46.4	4.6	9.0	45.9	8.6	14.7	45.8	4.1	4.7
	Q	31.9	6.6	16.4	28.4	2.7	4.7	27.9	5.4	9.6	27.9	2.6	3.2
4	P	50.7	11.0	23.0	46.4	4.7	9.1	45.9	8.6	14.8	45.9	4.1	4.7
	Q	31.9	6.7	16.4	28.4	2.7	4.8	27.9	5.4	9.7	27.9	2.6	3.2
Mean active power reduction (%)					8.40	57.93	60.76	9.34	21.84	35.84	9.44	62.29	79.45
Mean reactive power reduction (%)					10.99	59.84	71.25	12.55	18.93	41.28	12.55	60.60	80.42

It is observed in Table 4, Table 5 that the reduction of active and reactive power is different between the algorithms. For phase A, the loss reduction was similar between the studied algorithms. In phase B, the greatest loss reduction was obtained with NLogPSO, followed by EPSO and finally, APA. And in phase C, the most significant loss reduction was obtained by NLogPSO, followed by EPSO and APA. The reason is due to the dispersion of particles within their set (equivalent to the dispersion of particles within a cloud). Each algorithm has a distinct dispersion behavior given by its equation. Therefore, each algorithm makes its best current distribution between the branches, which directly affects the loss calculation. An important consideration is that the loads are unbalanced in the system and NLogPSO was the one that best distributed the currents in the branches.

**Fig. 5:**
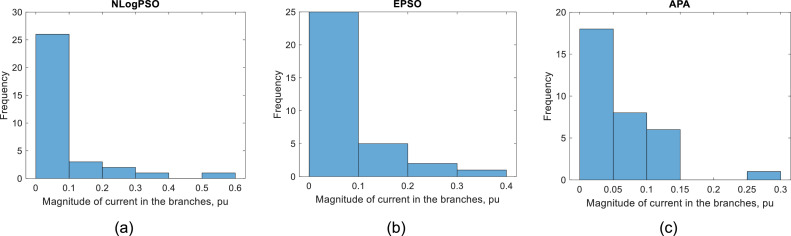
Histograms of the current magnitude in the branches at hour 1 by (a) NLogPSO, (b) EPSO and (c) APA.

**Fig. 6:**
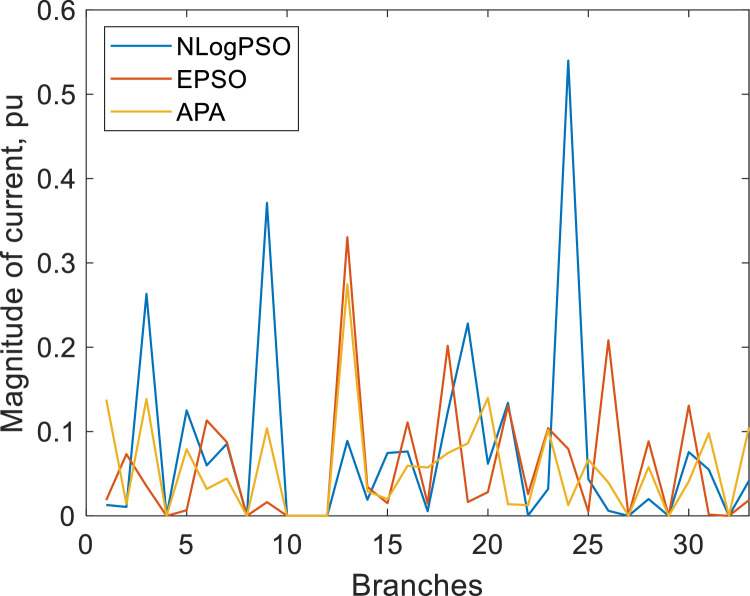
Comparison of the current magnitudes in the branches by NLogPSO, EPSO and APA algorithms.

Figs. 5 shows the histograms of the current magnitudes for hour 1 of the island system by the NLogPSO, EPSO and APA methods, respectively. Fig. 6 compares current magnitudes at hour 1 in the branches.

Loss reduction by NLogPSO optimization has the advantage of supplying a higher priority load. For example, considering hour 1, a 10 % reduction in phase A represents a saving of 5.05 kW, a 60 % reduction in phase B represents 6.48 kW and a 70 % reduction in phase C, 15.96 kW. The reduction in average percentage losses is equivalent to the optimization of power flow with and without harmonics by NLogPSO. The same is true for the APA and EPSO algorithms.

The DG optimal capacity was calculated with 100 particles in the second layer of the algorithm for EPSO and APA. Table 6 shows the values of each DG.

**Table 6:** Results of DG optimal capacity with EPSO and APA algorithms.

Algorithm	EPSO			APA and NLogPSO		
Hour	DG 1 (kW)	DG 2 (kW)	DG 3 (kW)	DG 1 (kW)	DG 2 (kW)	DG 3 (kW)
1	1208.1	54.7	80.5	1207.8	56.5	78.2
2	1176.1	53.3	76.4	1171.4	51.0	85.6
3	1280.0	54.7	61.6	1280.1	54.7	61.6
4	1281.7	51.6	60.2	1281.7	51.6	60.2

The computational time to compare the proposed algorithms considers the harmonic temperature-BFS, DG optimal allocation, island harmonic temperature-BFS and the optimization in 2 layers. APA-APA, EPSO-EPSO and NLogPSO algorithms’ computational time are 76.58 s, 21.49 s and 44.68 s, respectively. All algorithms responded with 1 run and 101 iterations.

For the APA-EPSO algorithm, the optimal values were obtained with *n*={2000,2500,2500} particles, where *n* represents the particle number used in each system phase, *n*={*n_A_, n_B_, n_C_*}. These particles were applied in the APA algorithm for loss minimization. Optimal $\lambda =\{\lambda_{A},\lambda_{B},\lambda_{C}\}$ for loss reduction was λ={19,25,25} and for optimal DG capacity was 2. Table 7, Table 8 present active and reactive power results with APA-EPSO optimization for wind speeds of 0.2 and 2.0 m s^-1^, respectively. The computional time of the APA-EPSO algorithm is 301.12 s. APA-EPSO showed a difference in the optimization of losses of 6.6 % in phases A and B, and 23.52 % in phase C compared to APA-APA and wind speed of 0.2 m s^-1^. And regarding reactive power, this difference was 38.97 % and 14.07 % in phases B and C, respectively. And for a wind speed of 2 m s^-1^, the difference in the APA-EPSO loss optimization concerning the APA-APA was 39.82 % and 19.42 % in phases B and C. And in the case of reactive power was 39.03 % and 14.50 % in phases B and C, respectively.

**Table 7:** Results of active and reactive power with harmonic temperature-BFS and APA-EPSO algorithms for the wind speed of 0.2 m s^-1^.

Hour	kW	Harmonic Temperature-BFS			APA-EPSO		
	kVAr	A	B	C	A	B	C
1	P	52.0	11.1	23.5	48.3	8.4	14.5
	Q	31.8	6.5	16.3	27.8	5.1	8.8
2	P	52.0	11.1	23.5	48.4	8.4	14.5
	Q	31.8	6.6	16.3	27.8	5.1	8.8
3	P	52.1	11.3	23.6	48.5	8.5	14.6
	Q	31.9	6.6	16.4	27.8	5.1	8.9
4	P	52.2	11.3	23.7	48.5	7.2	12.9
	Q	31.9	6.7	16.4	27.9	4.7	8.9
Mean active power reduction (%)					7.00	27.43	40.07
Mean reactive power reduction (%)					12.64	24.21	45.87

**Table 8:** Results of active and reactive power with harmonic temperature-BFS and APA-EPSO algorithms for the wind speed of 2.0 m s^-1^.

Hour	kW	Harmonic Temperature-BFS			APA-EPSO		
	kVAr	A	B	C	A	B	C
1	P	50.5	10.8	22.8	45.7	8.0	13.7
	Q	31.8	6.5	16.3	27.8	5.1	8.8
2	P	50.5	10.8	22.8	45.7	8.0	13.7
	Q	31.8	6.6	16.3	27.8	5.1	8.8
3	P	50.6	10.9	22.9	45.8	8.1	13.8
	Q	31.9	6.6	16.4	27.8	5.1	8.9
4	P	50.7	11.0	23.0	45.8	6.8	12.2
	Q	31.9	6.7	16.4	27.9	4.7	8.9
Mean active power reduction (%)					9.54	28.93	41.63
Mean reactive power reduction (%)					12.64	24.22	45.87

**Fig. 7:**
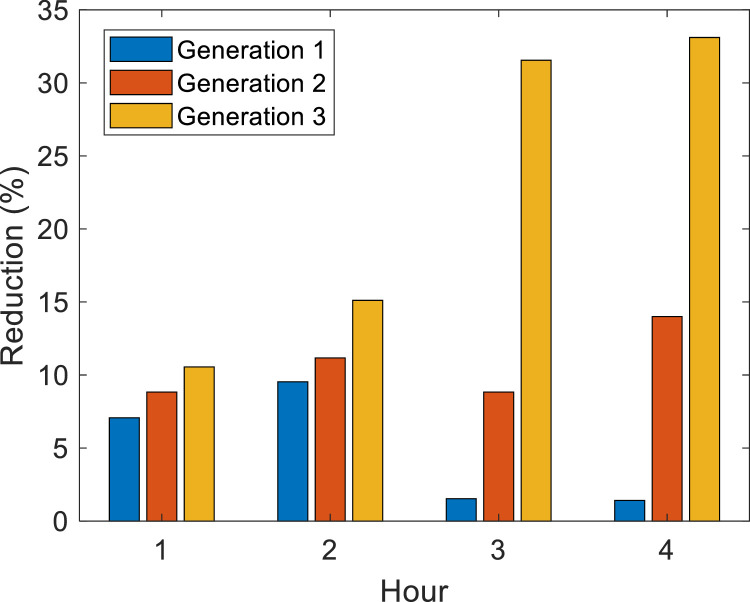
Percentage reduction of generation for case 1.

Fig. 7 shows the percentage reduction in each generation, which can be RMPS or DG, concerning its rated capacity. Generation 1 is at bus 890 with a rated capacity of 1300 kW, generation 2 is at bus 830 with a total of 60 kW, and generation 3 is at bus 844 with a 90 kW rated capacity.

For the EPSO-APA algorithm, the optimal values were obtained with *n*={2000,500,500} particles, where *n*={*n_A_, n_B_, n_C_*}, represents the particle number used in each system phase. These particles were applied in the APA algorithm for loss minimization. Optimal $\lambda =\{\lambda_{A},\lambda_{B},\lambda_{C}\}$ for loss reduction was λ={25,20,20} and for optimal DG capacity was 2. Table 9, Table 10 present the results of active and reactive power with EPSO-APA optimization for wind speeds of 0.2 and 2.0 m s^-1^, respectively. The computational time of EPSO-APA algorithm is 43.14 s. Loss reduction is similar between EPSO-APA and EPSO-EPSO.

**Table 9:** Results of active and reactive power with harmonic temperature-BFS and EPSO-APA algorithms for the wind speed of 0.2 m s^-1^.

Hour	kW	Harmonic Temperature-BFS			EPSO-APA		
	kVAr	A	B	C	A	B	C
1	P	52.0	11.1	23.5	48.9	4.8	9.8
	Q	31.8	6.5	16.3	28.3	2.7	6.3
2	P	52.0	11.1	23.5	48.9	4.8	9.9
	Q	31.8	6.6	16.3	28.5	2.7	6.4
3	P	52.1	11.3	23.6	49.0	5.0	10.0
	Q	31.9	6.6	16.4	28.6	2.8	6.4
4	P	52.2	11.3	23.7	49.0	7.4	10.0
	Q	31.9	6.7	16.4	28.6	4.4	6.5
Mean active power reduction (%)					6.00	50.94	57.90
Mean reactive power reduction (%)					10.52	52.36	60.86

**Table 10:** Results of active and reactive power with harmonic temperature-BFS and EPSO-APA algorithms for the wind speed of 2.0 m s^-1^.

Hour	kW	Harmonic Temperature-BFS			EPSO-APA		
	kVAr	A	B	C	A	B	C
1	P	50.5	10.8	22.8	46.1	4.6	9.3
	Q	31.8	6.5	16.3	28.5	2.7	6.3
2	P	50.5	10.8	22.8	46.2	4.6	9.3
	Q	31.8	6.6	16.3	28.5	2.7	6.4
3	P	50.6	10.9	22.9	46.3	4.7	9.4
	Q	31.9	6.6	16.4	28.6	2.8	6.4
4	P	50.7	11.0	23.0	46.3	7.0	9.5
	Q	31.9	6.7	16.4	28.6	4.4	6.5
Mean active power reduction (%)					8.60	52.01	59.02
Mean reactive power reduction (%)					10.36	52.36	60.86

**Fig. 8:**
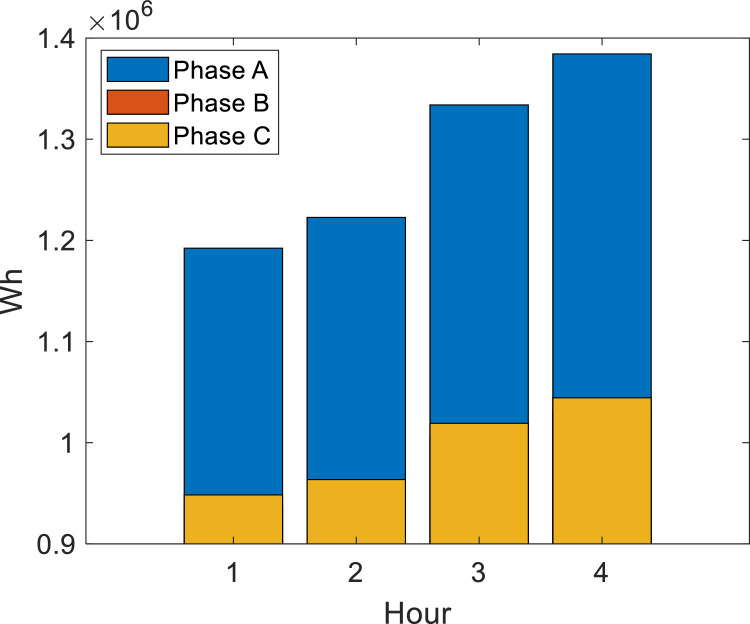
Load power demand for case 2.

For **case 2**, the maximum generation power of RMPS at bus 890 is 1300 kW per phase, 90 kW per phase at bus 830 and 200 kW per phase at bus 844 bus, *P_RMPS_* = {1300,90,200} kW in the buses *N_RPMS_* = {890,830,844}. Due to the skin effect, the induction motor is at bus 828 and influences the branch (824,828). Fig. 8 shows the load power demand during island operation for each phase.

For APA-APA, EPSO-EPSO and NLogPSO—NLogPSO algorithms, the optimal values were obtained with 1000 particles for the loss minimization in the harmonic temperature power flow. The optimal λ for losses reduction was 30 for APA-APA and NLogPSO—NLogPSO algorithms, and 25 for EPSO-EPSO. The optimal DG capacity was 2. Table 11 presents the average loss optimization in the 4 h of island mode when comparing the EPSO-EPSO, APA-APA and NLogPSO—NLogPSO algorithms with a harmonic temperature-BFS algorithm without optimization. The optimal DG capacity was calculated with 100 particles in the second layer of the algorithm for EPSO and APA. Table 12 shows the values of each DG.

**Table 11:** Average losses percentage reductions with EPSO-EPSO, APA-APA and NLogPSO—NLogPSO for the wind speeds of 0.2 m s^-1^ and 2.0 m s^-1^.

Algorithms	EPSO-EPSO			APA-APA			NLogPSO—NLogPSO		
Wind speed = 0.2 m s^-1^	A	B	C	A	B	C	A	B	C
Mean active power reduction (%)	2.15	31.51	56.18	2.80	14.76	26.32	2.79	70.45	73.67
Mean reactive power reduction (%)	3.65	32.21	58.10	6.31	3.80	27.68	6.31	56.25	75.06

Wind speed = 2.0 m s^-1^	A	B	C	A	B	C	A	B	C
Mean active power reduction (%)	4.87	33.66	57.19	5.59	18.79	28.26	5.53	68.99	74.21
Mean reactive power reduction (%)	3.65	31.55	58.35	6.31	5.11	27.68	6.30	52.31	75.06

**Table 12:** Results of DG optimal capacity with EPSO and APA algorithms.

Algorithm	EPSO			APA or NLogPSO		
Hour	DG 1 (kW)	DG 2 (kW)	DG 3 (kW)	DG 1 (kW)	DG 2 (kW)	DG 3 (kW)
1	1196.7	73.4	196.6	1192.4	86.9	156.6
2	1223.3	86.8	177.0	1223.3	76.7	165.1
3	1259.1	77.9	184.2	1259.1	84.3	177.6
4	1259.1	76.5	189.4	1259.1	84.5	146.7

The processing time of the algorithm considers harmonic temperature-BFS, optimal DG allocation, island harmonic temperature-BFS and the optimization in 2 layers. The computational times are 81.30 s, 31.95 s and 36.48 s for APA-APA, EPSO-EPSO and NLogPSO—NLogPSO algorithms, respectively.

For the APA-EPSO algorithm, the optimal values were obtained with *n*={2000,2500,2500} particles. These particles were applied in the APA algorithm for loss minimization. Optimal $\lambda =\{\lambda_{A},\lambda_{B},\lambda_{C}\}$, λ={19,25,25} for losses reduction, and optimal DG capacity was 2. The computational time of the APA-EPSO algorithm is 213.23 s.

For the EPSO-APA algorithm, the optimal values were obtained with *n*={2000,500,500} particles, where *n*={*n_A_, n_B_, n_C_*}, represents the particle number used in each system phase. These particles were applied in the APA algorithm for loss minimization. Optimal λ={25,20,20}for losses reduction, and optimal DG capacity was 2. The computational time of the EPSO-APA algorithm is 40.72 s. Table 13 presents the average loss optimization in the 4 h when comparing the APA-EPSO and EPSO-APA algorithms with a harmonic temperature-BFS algorithm without optimization.

**Table 13:** Average losses percentage reductions with APA-EPSO and EPSO-APA for the wind speeds of 0.2 m s^-1^ and 2.0 m s^-1^.

Algorithms	APA-EPSO			EPSO-APA		
Wind speed = 0.2 m s^-1^	A	B	C	A	B	C
Mean active power reduction (%)	1.94	20.50	28.07	3.23	36.07	47.03
Mean reactive power reduction (%)	5.57	11.54	26.92	6.94	30.43	50.13

Wind speed = 2.0 m s^-1^	A	B	C	A	B	C
Mean active power reduction (%)	4.71	23.35	29.91	6.37	37.68	48.43
Mean reactive power reduction (%)	5.57	11.54	26.92	8.04	30.44	50.62

**Fig. 9:**
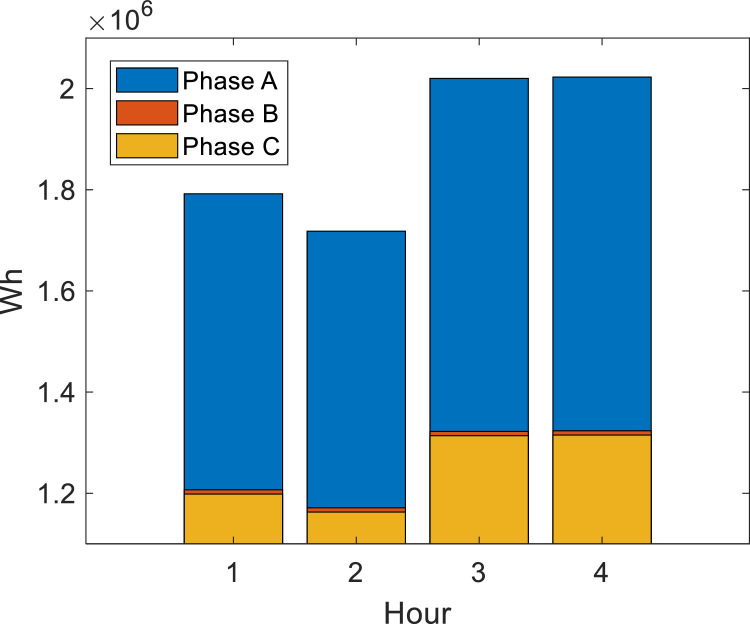
Load power demand for case 3.

For **case 3**, the maximum generation power of RMPS at bus 890 is 1300 kW per phase, at bus 830 is 500 kW per phase, at bus 844 is 500 kW per phase and at bus 860 is 200 kW per phase, *P_RMPS_* = {1300,500,500,200} kW in the buses *N_RPMS_* = {890,830,844,860}. Due to the skin effect, the induction motor is at bus 828 and influences the branch (824,828). Fig. 9 shows the load power demand during island operation. For APA-APA and EPSO-EPSO algorithms, the optimal values were obtained with 1000 particles for loss minimization in the harmonic temperature power flow. Optimal λ for loss reduction was 17 for the APA-APA algorithm and 7 for EPSO-EPSO. The optimal DG capacity was 2. Table 14 presents the average loss optimization in the 4 h when comparing the EPSO-EPSO and APA-APA algorithms with a harmonic temperature-BFS algorithm without optimization.

**Table 14:** Average losses percentage reductions with EPSO-EPSO and APA-APA for the wind speeds of 0.2 m s^-1^ and 2.0 m s^-1^.

Algorithms	EPSO-EPSO			APA-APA			NlogPSO—NlogPSO		
Wind speed = 0.2 m s^-1^	A	B	C	A	B	C	A	B	C
Mean active power reduction (%)	12.41	26.91	68.19	12.00	33.84	43.40	12.00	54.68	70.48
Mean reactive power reduction (%)	17.52	30.87	71.55	15.81	35.27	46.50	15.81	53.56	74.15

Wind speed = 2.0 m s^-1^	A	B	C	A	B	C	A	B	C
Mean active power reduction (%)	14.77	28.98	69.05	14.38	35.68	45.00	14.38	55.68	71.40
Mean reactive power reduction (%)	17.52	30.74	71.55	15.81	35.12	46.50	15.81	53.46	74.15

**Fig. 10:**
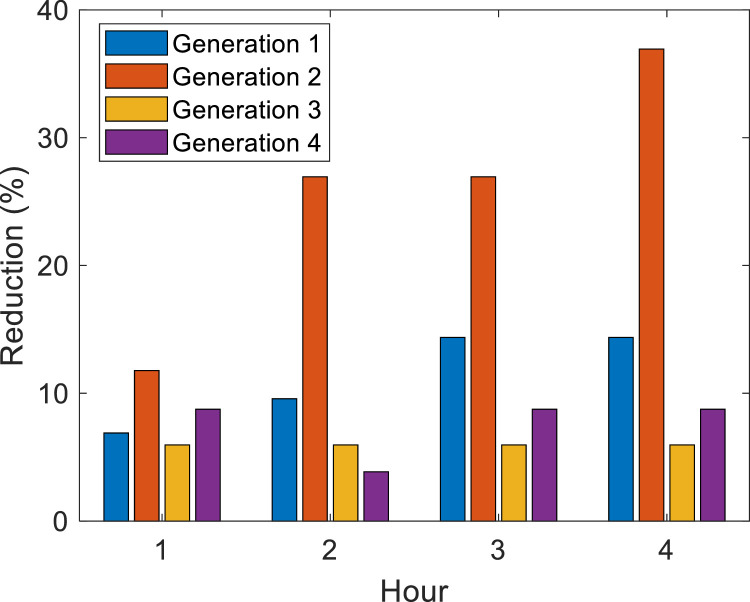
Percentage reduction of generation for case 3.

The optimal DG capacity was calculated with 100 particles in the second layer of the algorithm for EPSO and APA. Fig. 10 shows the percentage reduction in each generation, which can be RMPS or DG, in relation to its nominal capacity. Generation 1 is at bus 890 with a rated capacity of 1300 kW, generation 2 is at bus 830 with a rated capacity of 500 kW, generation 3 is at bus 844 with a 500 kW rated capacity and generation 4 is at bus 860 with a 200 kW rated capacity. The processing time of the algorithm considers the harmonic temperature-BFS, DG optimal allocation, island harmonic temperature-BFS and the optimization in 2 layers.

For the APA-EPSO algorithm, the optimal values were obtained with *n*={2000,2500,2500} particles. These particles were applied in the APA algorithm for loss minimization. Optimal *λ*={17,17,17} for loss reduction and the optimal capacity of DG was 2. For the EPSO-APA algorithm, the optimal values were obtained with *n*={2000,500,500} particles. These particles were applied in the EPSO algorithm for loss minimization. Optimal *λ*={10,10,10} for losses reduction and the optimal DG capacity was 2. Table 15 presents the average loss optimization in the 4 h when comparing the APA-EPSO and EPSO-APA algorithms with a harmonic temperature-BFS algorithm without optimization.

**Table 15:** Average losses percentage reductions with APA-EPSO and EPSO-APA algorithms for the wind speeds of 0.2 m s^-1^ and 2.0 m s^-1^.

Algorithms	APA-EPSO			EPSO-APA		
Wind speed = 0.2 m s^-1^	A	B	C	A	B	C
Mean active power reduction (%)	12.00	37.06	39.06	11.26	19.86	58.80
Mean reactive power reduction (%)	15.81	37.28	41.31	15.87	23.88	63.65

Wind speed = 2.0 m s^-1^	A	B	C	A	B	C
Mean active power reduction (%)	14.38	38.84	40.77	13.58	21.87	59.95
Mean reactive power reduction (%)	15.81	37.14	41.42	15.87	23.71	63.77

### Method validation II: considerations about priority load

**Fig. 11:**
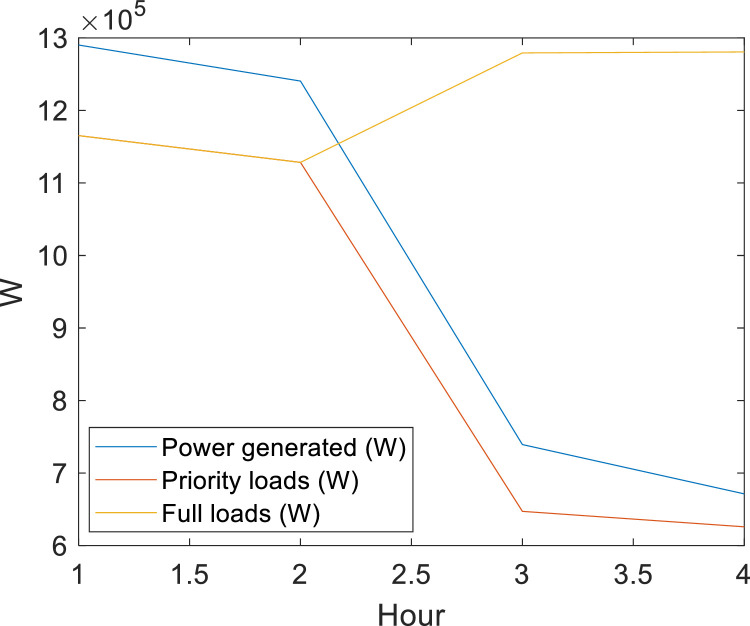
Comparison of priority loads and full loads with the power generated in the period of 4 h.

The energy supplied by the RMPS may decrease with time, considering that the generation can supply its rated capacity in the first and second hours, and supplies 75 % and 50 % of its capacity in the third and fourth hours, respectively. The first disconnected loads are at buses with *l* = 3 (those with the lowest priority), which in the case studied are $N_{B}^{\langle 3\rangle }=\left\{818,820,822,848,840,834,844,816,824,890,858,810\right\}$ and the second disconnected loads are $N_{B}^{\langle 2\rangle }=\left\{828,830,832,858,860,864\right\}$. Fig. 11 shows that generation optimization guarantees the priority loads previously defined with APA, EPSO or NLogPSO algorithms. In this case, it is observerd that, without adjusting loads with generation, the system would only be able to supply its energy for 2 h.

In this case, with a VFD at bus 816, priority *l* = 3, and an induction motor at bus 828, priority *l* = 2. The difference in system losses with and without harmonics results in an average of 1.679 kW, 5.38 kW and 5.508 kW in phases A, B and C, respectively.

If a VFD is added at bus 850, with priority *l* = 1, in this case, there are three harmonic sources: two VFDs (buses 816 and 850) and an induction motor (bus 828). The difference in system losses with and without harmonics results in an average of 3.226 kW, 7.187 kW and 7.076 kW in phases A, B and C, respectively. Thus, comparing the two cases, there is an increase of 92.13 %, 33.58 % and 28.46 % in phases A, B and C, respectively.

Optimization of priority loads can be carried out by applying the NLogPSO algorithm. In this case, it is necessary to define the loads that can be disconnected and those that cannot. Thus, for the 34 Bus System, it was defined that the loads in the branches can remain disconnected with the reduction in the energy supplied by the DGs, which in the proposed scenario should start from hour 3 of the island mode. Thus, these loads that can remain disconnected are at buses *N*={810, 818, 820, 822, 826, 838, 846, 848, 856, 862, 864, 888} and make up the optimization constraint. Another constraint is the load that must remain connected during the entire island mode, and in this case, it is at bus 890.

**Fig. 12:**
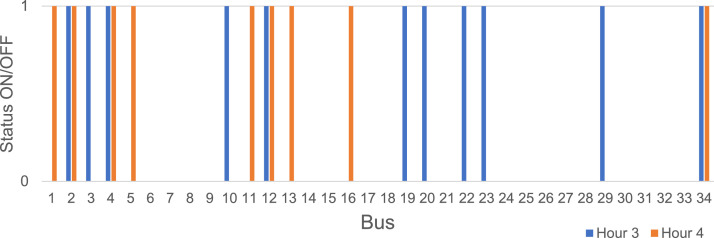
Loads connected at hours 3 and 4.

The state of the loads is indicated by the particle that assumes the value 1 and, off, 0. The loads connected at hours 3 and 4 are shown in Fig. 12, where the original numbering of the system is in Table 16. Therefore, NLogPSO algorithm also can be applied to guarantee the supply of the loads indicated by the optimization.

**Table 16:** Original numbering of the system.

Original Bus	Renamed Bus	Original Bus	Renamed Bus	Original Bus	Renamed Bus	Original Bus	Renamed Bus
800	1	824	10	850	19	826	28
802	2	828	11	852	20	840	29
806	3	830	12	854	21	848	30
808	4	832	13	858	22	856	31
812	5	834	14	860	23	864	32
814	6	836	15	862	24	838	33
816	7	842	16	888	25	890	34
818	8	844	17	810	26		
820	9	846	18	822	27		

### Summary comments

This paper presented the two layer NLogPSO algorithm to solve the optimization of losses and energy management in the IEEE-34 unbalanced island system. NLogPSO algorithm presents the behavior of the particle based on the natural logarithm and has been compared with the two-layer algorithms: APA-APA, APA-EPSO, EPSO-EPSO and EPSO-APA. In terms of reducing losses in the island system, NLogPSO obtained the best result in all scenarios, followed by EPSO-EPSO and APA-APA. The average active power reduction with NLogPSO in phase A was 6.86 %, 2.79 % and 12.0 % for cases 1, 2 and 3 respectively and for reactive power 12.56 %, 6.31 % and 15.81 %. For phase B, the average active power reduction was 61.60 %, 70.45 % and 54.68 % for cases 1, 2 and 3 respectively, and for reactive power 60.60 %, 56.25 % and 53.56 %. Finally, for phase C, the average active power reduction was 79.0 %, 73.67 % and 70.48 % for cases 1, 2 and 3 respectively, and for reactive power 80.42 %, 75.06 % and 74.15 %. There was an approximate reduction in losses between the scenarios tested, where the installed power was 1.45 MW, 1.59 MW and 2.50 MW for cases 1, 2 and 3 respectively. Depending on the reduction in losses, it is possible to feed another load, or it may indicate an increase in generation autonomy to supply energy to the system. Thus, the importance of studying the losses of an island system is justified. Another critical point is the definition of priority loads in the optimization process. This is because this way it is possible to extend the operating time of the island system and improve energy management.

The proposal also involved optimal particle initialization to reduce losses and generation capacity, since it is a necessary part of the convergence of particle-based algorithms. The Eqs. (24) and (25) proposed for the initial particles define an initial search space for the NLogPSO, APA and EPSO algorithms. In the case of NLogPSO, the calculation of the initial particles is aided by the l and determines the logarithmic trajectory within the solution set. To determine the optimum capacity of the RMPS, the l was set to 2 for all scenarios and all algorithms. For loss reduction, the l was 20, 30 and 17 for cases 1, 2 and 3 respectively using the NLogPSO algorithm and 1000 particles.

The application of particle-based algorithms showed good performance when using the current in the branches as particles in the radial configuration. Regarding the classic PSO, it was not discussed in this paper, as it did not achieve convergence for the problem studied. A proposal for future work is to evaluate NLogPSO in other configurations and reconfigurations of power systems. There is also the study of the impact of harmonics on an island system and how this can affect the autonomy of the energy supply.

## Ethics statements

Not applicable

## CRediT authorship contribution statement

**Alessandra F. Picanço:** Conceptualization, Methodology, Validation, Investigation, Writing – original draft. **Antônio C. Zambroni de Souza:** Conceptualization, Methodology, Investigation, Validation, Visualization, Supervision, Writing – review & editing. **Andressa Pereira Oliveira:** Visualization, Writing – review & editing.

## Declaration of competing interest

The authors declare that they have no known competing financial interests or personal relationships that could have appeared to influence the work reported in this paper.

## Data Availability

Data will be made available on request.
